# Understanding Male Caregivers’ Emotional, Financial, and Physical Burden in the United States

**DOI:** 10.3390/healthcare7020072

**Published:** 2019-05-22

**Authors:** Monika Lopez–Anuarbe, Priya Kohli

**Affiliations:** 1 Economics Department, Connecticut College, New London, CT 06320, USA; 2 Mathematics and Statistics Department, Connecticut College, New London, CT 06320, USA; pkohli@conncoll.edu

**Keywords:** men caregivers, caregiver burden, unpaid caregiving, family caregiving, caregiver support

## Abstract

Men caregivers face caregiving burden, have weak support networks and are less likely to seek out programs which increase their caregiving capabilities and help them cope with this burden. Using the 2011 and 2015 National Study of Caregiving (NSOC) database and hierarchical regressions, we studied the emotional, financial, and physical burden of male caregivers as spouses, sons, and other caregivers by assessing the impact of caregiver characteristics, tasks and resources for each subgroup. We highlighted the importance of using a nationally representative database for men caregivers only and emphasized that these caregivers are not a monolithic group. We found that all caregivers experienced these three burden types, particularly elevated emotional stress, with sons reporting the highest emotional and financial strain levels. Assisting with personal care was the most stressful task and caregivers vastly under-utilized support and training. Our results suggest that burden suppressants included having family and friends help with caregiving, having time to decompress, and feeling appreciated by the care recipient. These findings offer insight for devising future policies that intentionally include relationship and burden type to encourage improved and more caregiving from men while supporting their well-being.

## 1. Introduction and Background Literature

In the United States, unpaid family members are the largest group of caregivers assisting an aging person, followed by home care aides and certified nurse assistants [1]. The potential pool of family caregivers is decreasing rapidly [2], thus there is a need to create more effective policies which provide better support to these caregivers and that attract new ones. One possible solution is to encourage more men into the caregiving pool as this percentage is increasing, anyway, currently estimated at 40% of all informal caregivers, or about 16 million men [3]. 

Out of these 16 million male caregivers, about half decided to assist their loved one(s) by choice and 63% identified as primary caregivers. On average, they provided care for nearly four years (spouses for five years), and 52% thought that they would care for someone in the next five years [3]. 49% were assisting an aging parent/in-law and 13% a spouse; 59% were white, 13% black, 7% Asian American, and 19% Hispanic; in the latter case, 32% of millennial caregivers identified as Hispanic. Regarding income, 44% of households were below $50,000 even though they were more likely than female caregivers to work outside the home [4,5,6,7,8,9,10,11], and 56% were married, while 11% identified as gay, bisexual, or transgender [3,12]. 

We contribute to the existing literature by studying two years (2011 and 2015) of a nationally representative dataset (the National Study of Caregiving) unlike most of the existing work that has used small or non-random sample sizes, compares male and female caregivers, studies one type of male caregiver, or treats men caregivers as a monolithic group behaving homogeneously and experiencing caregiving in the same way. We believe that factors such as caregiver relationship are just as important as gender in explaining these experiences, and explored how these three burden types depended on the caregiver relationship with their recipient (as husbands or partners, sons, and other caregivers such as: sons-in-law, grandsons, brothers and friends), caregiver characteristics (including work status, relationship, enough time for himself and feels appreciated), caregiving frequency (number of caregiving hours, giving care for over an year, how often they helped with specific tasks like personal care), and resources (including family and friends assisting, received training and information). A more detailed explanation of our contribution is in Section 1.7.

### 1.1. Why do Men Care?

Some authors [4,5,6,7,8,9,10,11] explain why men choose to or end up caring for another adult given the historical connotation of caregiving as “women’s work”, and the “feminine” nature of many caregiving tasks [5,6,11]. Hanlon [9] identifies three configurations of masculinities related to caring and work: conventional, sharing, and caring masculinities. Conventionalists relate their masculinity through paid work and are not conflicted to sacrifice paid work since they do not have caring responsibilities or because the women in their lives do the caring work. Sharers, instead, have a strong sense of caring identity and responsibility, and are involved in all aspects of caring. They still work outside of the home and struggle balancing paid work with caring responsibilities to maintain a sense of themselves as men. Finally, carers do not define masculinity through paid work even if they work outside the home, have a strong sense of caring identity and caring responsibility, and mostly define caring as nurturing rather than task completion. 

Caring masculinities help explain why men decide to—or not to—provide assistance to a loved one, since they refer to the notion that masculine identities exclude domination and embrace the affective, relational, emotional, and interdependent qualities of care [7], thereby rejecting hegemonic masculinity [4,7,8] and integrating the values and practices of care into their masculine identities [8]. Care work could also be congruent with patriarchal ideals of gender roles if the emotional involvement is replaced with the roles of provider, protector, and problem solver. In the end, what matters is that men get involved in caregiving roles regardless of their motives [10]. 

### 1.2. How do Men Care?

Researchers acknowledge significant differences in caring patterns, burden, and responses to caregiving between men and women caregivers [11]. For example, male caregivers may sometimes have a more task-oriented approach to care, accessing fewer formal services and support whereas female caregivers may use more emotion-oriented coping methods [6,11,13,14]. Care is also structured along roles [11,15,16]. In the United States, participating in the labor market can help men practice their masculinities, turning the caregiving role into a job that requires solving challenging problems to achieve concrete goals [16,17]. 

Gender is only one facet of a caregiver’s identity; how it combines with other factors and roles affects caregiving style, tasks, and experience [18]. Most research on family caregiving has historically focused on female assistance, so existing policies supporting caregivers were typically aimed at women and how they cared for a loved one. This raises the question of whether “best practices” for caregiving are measured with a female yardstick: men’s contributions to long-term caregiving may have been marginalized and under-estimated [19], especially when some men do not even label themselves as caregivers despite performing tasks that would characterize them as such and, instead, identify themselves as a relative of the care recipient, such as husbands tending for their wives [11,20,21]. 

### 1.3. Men Caregivers and Burden

A caregiver’s relationship status with their recipient, the caregiving intensity and the types of performed tasks impact their burden and self-esteem. For example, even though daughter caregivers experienced a higher burden level than son caregivers, sons reported lower self-esteem in some cases [22]. Sons and daughters who are caregivers experience considerable emotional burden in situations where the care recipient has moved into their home and has behavioral problems, or where the caregiver needs to work outside the home to make ends meet [16]. In fact, younger caregivers reported more emotional stress when having to choose between work and caregiving responsibilities [3]. Likewise, stress is a significant issue for husband caregivers [11,23] and these caregivers, despite experiencing high levels of physical stress, report low levels of caregiver burden [24]. Compared to other family male caregivers, husbands provide more hours of care, tend to be the primary caregivers, and 78% have little to no support from other caregivers [3]. Hence, spouses represent a group with caregiving characteristics distinct from adult children and other family members, so it is incorrect to generalize research findings from mixed samples [16,25]. 

Though an earlier body of literature documented that, compared to women caregivers, men faced lower levels of depression, strain, and psychological distress, other studies have disputed these findings [26,27,28,29]. Men may underreport the level of burden they experience because they are less likely to admit their negative feelings [26,30] or less in tune with how to process and share emotions [28,29,31], because existing burden instruments may not accurately speak to men’s experiences of burden [11], or because acknowledgment of burden could be culturally unacceptable [16]. Regardless of whether the literature favors one side or the other, or if men downplay their burden when answering these surveys, they do experience caregiver burden and self-esteem issues [30,32,33]: 62% reported moderate to very stressful caregiving experience and 46% moderate to severe physical strain [3].

### 1.4. Caregiving Tasks, Burden, and Support

While research is mixed on whether male caregivers help with tasks considered less nurturing and more specific [34,35], others [36,37] believe that caregiving responsibilities are now less gendered. In reality, some tasks are more emotionally, financially, or physically taxing for any caregiver irrespective of their gender or relationship [38], but additional factors determine the burden intensity that men caregivers face. For example, assisting with personal care activities such as taking their loved one to the toilet and bathing them were considered the most difficult tasks for all caregivers regardless of gender [12]. Male caregivers reported greater discomfort and difficulty, possibly because they had less experience performing these activities [3,39]. In fact, according to the 2015 National Study on Caregiving (NSOC), 54% of male participants found it moderately to very difficult to help recipients with their personal care needs [40], which may also help explain why they spend fewer hours assisting with personal care [11,41,42]. 

Regardless of the burden and discomfort, male caregivers are increasingly performing personal care and medical tasks such as: giving injections, tube feedings, catheter care, and other complex responsibilities like interacting with providers, agencies, and professionals as advocates and communicators for their care recipients. Over half of them indicated that they wanted a qualified person to show them how to do these tasks, and only 14% reported any preparation and training [3,12]. In fact, most caregivers lack adequate training or support, so it is urgent to improve their skills and support networks to diminish burden and increase competence [1]. Similarly, 84% of caregivers stated that they could use more information or help on caregiving topics, especially about keeping their loved one safe at home, managing their own stress, dealing with their care recipient’s challenging behaviors and with incontinence or toileting problems [12]. In fact, according to the AARP (American Association of Retired Persons), only one-third of family caregivers reported having a conversation with a medical or social work team member about what was needed to care for their relative, and nearly half reported receiving no training in wound care, one of the most difficult tasks they faced [43]. 

Caregivers often need supplementary companion care, respite care, help with household chores, hands-on care, meal preparation guidelines, and want to work more closely with home health aides to learn from them about providing person-centered care [44]. The success of mastering caregiving stress depends on the use of available physical resources and ways of coping with emotional demands [15,16]. Even if caregivers have to provide care despite not choosing to do so, supportive services may help mitigate some of the experienced burden that arises from complex care situations [12]. Also, the impact of fighting social isolation and investing in self-care play an important role, as larger family support networks, greater family agreement, and greater management of self-efficacy are associated with lower burden levels [45]. 

### 1.5. On Service (under) Utilization

Male and female caregivers underutilize any formal or informal support systems. Very few have used supportive services such as respite care (15%), transportation (23%), or financial assistance (28%), mostly due to inaccessibility and unaffordability [12]. Spousal caregivers experience more burden and depression than other family members, and their well-being and self-efficacy are lower [46]. They are much less demanding of services for their families and themselves compared to their sons, as can be confirmed with their significantly lower service usage [47]. Across all ethnicities, spouses gave care for more hours and performed more tasks than other family members [48] and were most reluctant to hire professional helpers or use community services [49]. This reluctance to delegate tasks or use outside services rendered them vulnerable to exhaustion and distress [50,51,52]. Consequently, male and female spouses report more serious issues around their ability to fulfill their caregiving role and the most negative health effects [53]. Their emotional burden is also compounded because they need to cope with the extra anxiety of possibly losing their relationship with their spouse and partner [25]. 

Some men are even less likely than women to use these caregiver support systems [19] and the additional non-gender-based reasons described above may reinforce this reluctance. Another possible explanation for this reluctance is linked to the constraints of holding traditional masculine values, as can be seen with Hanlon’s conservative masculinities [30], which may also inhibit men from reporting burden [11]. Moreover, a lack of orientation to caregiving, insufficient information, inadequate links to support resources, ineffectual communication, and hurtful interactions with support services impede effective caregiver support utilization, especially in rural areas, thereby leading to increasing isolation [11,54,55]. Men could also be less likely to use informal resources because most have smaller social networks than women and thus, more limited access to support [56]. Sometimes, this lower rate is more associated with inadequate knowledge than with resistance for getting support [57], so it is important for caregivers to know about these services and to find them valuable.

### 1.6. The Stress Appraisal Model

One relevant theoretical framework of caregiving that explain burden the way we do is the stress-appraisal model [45,58]. This model relates how caregivers appraise stressful situations with their reactions to them, and how coping with available resources may affect caregiving outcomes. The model has five components: need for care; an immediate judgment of care needs manifested in the care provided; personal and social resources altering the effect of stressors, such as: formal help, relationship quality, emotional support, and mastery; caregiver appraisal of the caregiving situation manifested as burden; and outcome or caregiver well-being.

Based on the stress-appraisal model and our own work, we hypothesize that all family caregivers experience caregiving burden, but that these burden manifestations are a function of caregiver relationship in addition to personal characteristics and caregiving resources or support. If the caregiver feels that his personal and family relationship with the recipient is positive and in line with his caregiver duties, family roles, and available resources, his burden will be easier to bear and may be lower. To identify predictors which can help explain caregiving burden, we used variables from the relevant existing literature (details below Section 2.2) and their statistical significance in explaining the odds of experiencing such strain. 

### 1.7. Our Study and Contribution

It is difficult to capture the considerable effort, commitment, and burden associated with this role [1]. Moreover, caregiver gender comparisons can sometimes inadvertently assume that one gender behaves uniformly, thereby ignoring the diversity and efforts of many who do not conform to common gender norms and do things their way [11]. In addition, most of the existing work has used small or non-random sample sizes, compares male and female caregivers, studies one type of male caregiver, or treats men caregivers as a monolithic group behaving homogeneously and experiencing caregiving in the same way. We believe that factors such as caregiver relationship are just as important as gender in explaining these experiences. Therefore, to provide a richer and more realistic picture, it is important to study the differences among male caregivers’ burdens and experiences based on their personal characteristics and their relationship to the aging relative or friend. To this end, we drew on the National Study of Caregiving (NSOC) to examine the emotional, financial, and physical burden that male caregivers faced in United States in 2011 and 2015. We explored how these three burden types depended on the relationship with the care recipient (as husbands or partners, sons, and other caregivers such as: sons-in-law, grandsons, brothers and friends), caregiver characteristics (including work status, relationship, enough time for himself and feeling appreciated), caregiving frequency (number of caregiving hours, giving care for over a year, how often they helped with specific tasks like personal care), and resources (including family and friends assisting, received training and information). Our work is based on the theoretical framework from the stress-appraisal model [58]. We conjecture that caregiver relationship, sense of appreciation, overall caregiving frequency or intensity, and available support are significantly associated with higher or lower likelihoods of emotional, financial and physical burden.

## 2. Materials and Methods

### 2.1. Dataset

The National Study of Caregiving (NSOC) is a supplement to the National Health and Aging Trends Study (NHATS). NSOC respondents are family caregivers and unpaid unrelated helpers aiding with mobility, self-care, household activities, transportation, or medically oriented tasks. Through a telephone interview, caregivers were asked about the provided types, duration, and intensity of care, how it affected them (emotionally, physically, and financially), and the support services that they used.

Our work is based on this nationally representative dataset with a random sample of 2,007 caregivers in 2011 and 2,204 in 2015 [40]. NSOC is not longitudinal, as individual caregivers from 2011 were not necessarily re-interviewed in 2015, thus precluding us from following specific caregiver’s overtime. The study includes nine sections covering different aspects of caregiving, and raw observations were weighted to account for the sampling design. Men represented about one-third of the sample in each year (673 in 2011 and 718 in 2015). We divided this subsample into three groups: husbands/spouses/partners (26% in 2011 and 28% in 2015), sons (54% in 2011 and 53% in 2015), and “others” (21% in 2011 and 20% in 2015). The “others” caregiver group included brothers, cousins, fathers, friends, grandsons, nephews, and sons-in-law. We used the terms husband, partner, and spouse indistinctively.

### 2.2. Study Variables

The NSOC database has 298 questions. We first identified a subset of 122 variables on how overwhelmed male caregivers were, controlling for missing values, non-responses, and responses such as “not applicable” or “I don’t know.” Our study variables were based on the following separate binary question(s) on emotional, financial, or physical difficulty experienced by the caregiver when helping the recipient (sample person, SP): “Is helping SP emotionally/financially/physically difficult for you?” Like other authors [22,46], we focused on reported emotional, financial, and physical difficulty. From the remaining variables, we created 12 categories, including: caregiver demographics, frequency of help, personal help, medical help, help with bills or banking, financial and employment status of caregiver, emotional status of caregiver, caregiver looking for support, support from family or friends, support from other resources, physical health, and other caregiver difficulties. 

We identified 18 predictor variables and then classified them into three primary categories: caregiver characteristics, caregiving tasks and frequency, and caregiver resources. Caregiver characteristics included seven predictors: age, marital status, education, employment, feeling appreciated, had time for himself, and general health status. Caregiving tasks and provided care frequency had eight predictors: assisting with personal care and house chores, helped speaking to a medical provider [59,60]; whether the caregiver helped longer than a year, number of hours assisted each day; helping with bills or banking transactions [61,62]; tasks which improve elder mobility and require physical effort from the caregiver such as, helping the person get around a room and leaning for support [63,64]. Finally, the caregiver resources category added three predictors: family and friends who can aide him, if the caregiver looked for help, and received caregiver training [1,19].

### 2.3. Methodology

To find out if caregiver relationship (spouse, son or other) impacted the reported emotional, financial, and physical burden, we used logistic regression models on each burden (“Is helping SP emotionally/financially/physically difficult for you?”) as a (binary) response variable and caregiver relationship as the (dummy) predictor variable. After noticing a significant association between burden and caregiver relationship, we separated each caregiver group. As a preliminary step, we used basic descriptive statistics (percentages for categorical variables and means/standard deviations for numerical variables) to study variability among spouses, sons and other caregivers. 

To study how caregiving impacted the odds of experiencing emotional, financial, and physical burden among spouses, sons and other caregivers, we used hierarchical logistic models. These models were logistic regressions since the response (“Is helping SP emotionally/financially/physically difficult for you?”) was binary. Hierarchical regressions involved the sequential addition of predictor variable blocks, which allowed us to examine the contribution of a particular block on the response beyond previously entered ones [65,66,67,68] and to explore how this contribution changed when new block(s) were added. Before running regression models, we tested for assumptions of the logistic model. Our response variable(s) were binary, observations were independent as they were from different caregivers, and data from 2011 and 2015 was not longitudinal. We then tested for the multicollinearity among predictor variables in the three categories. Since predictor variables (except number of hours helped per day) were categorical, we employed Pearson’s chi-squared test [69] and found several significantly correlated predictor variables. To alleviate multicollinearity issues, we removed the highly correlated variables from our three categories. From the remaining uncorrelated variables, we created three blocks. Block 1 consisted of three binary variables: 1. Have you worked for pay last week? 2. Do you have enough time for yourself? 3. Do you feel that the elderly appreciates you? Block 2′s variables were: 1. How often do you provide personal care? (every day; most days; some days; rarely and never)? 2. Number of caregiving hours provided each day? 3. Have you helped with caregiving for longer than a year? The final block of support availability and utilization for caregivers also had three (binary) variables: 1. Do you have friends and family to help with caregiving? 2. Have you ever looked for help? 3. Did you receive training or help? We summarize the predictor variables included in each of the three blocks below in Table 1. We found an approximate linear relationship among these predictor variables and log odds. Thus, the response variable(s) and selected predictor variables satisfied required assumptions for the logistic regression model.

## 3. Results

Logistic regression models provided the results in the form of expected changes in the log odds of burden when caregiver relationship changed. Our study, with the caregiver relationship as a categorical predictor for each individual burden (Table 2) showed that, compared with other caregivers, sons reported much higher and statistically significant log odds (0.88 in 2011 and 0.72 in 2015) of experiencing emotional stress, followed by spouses (0.34 in 2011 and 0.29 in 2015) compared to other caregivers. In addition, sons experienced the highest log odds of financial burden (especially in 2011) whereas husbands were less likely to report financial stress compared to sons and other caregivers, as reflected by the small negative sign of this coefficient. However, among all relatives, husband caregivers showed the highest (and most statistically significant) log odds for experiencing physical stress. 

Among all caregivers, we found that partners, sons, and others reported higher emotional burden (32%, 46% and 25% in 2011; 30%, 39%, and 24% in 2015) compared to financial (19%, 27% and 18% in 2011; 15%, 17%, and 16% in 2015) and physical strain (20%, 18% and 14% in 2011; 18%, 13%, and 11% in 2015), even though all caregivers reported high levels of care recipient appreciation (close to 88%). When asked if they had enough time for themselves, sons reported the lowest overall percentages (57% in 2011 and 52% in 2015), then other caregivers (66% in 2011 and 60% in 2015) and husbands (72% in 2011 and 50% 2015). The disparaging percentage difference of husbands between years is noteworthy. 

For the predictor variables in the caregiver characteristics category, husbands/partners had an age range of 65 to 89 years, sons’ age ranges were 25 to 64, and others had the widest age range of 20 to 80 years. Since age varies significantly with caregiver relationship, we also compared the change in burden (emotional, financial, and physical) based on relationship-controlling for caregiver age- and found that, compared to caregiver relationship, age ranges were an explanatory factor for the variation in all three types of burden. About half of sons and other caregivers were married (50% in 2011 for both; 49% in 2015 for sons and 46% for others), approximately one-seventh of sons were divorced (13% in 2011 and 15% in 2015), and other caregivers had a significantly higher divorce rate in 2011 (29% compared to 12% in 2015). At least half of each caregiver group had some college background in 2011 (58% of sons, 52% of husbands, and 50% of other caregivers), with a lower percentage of husbands (44%) in 2015 and comparable values for the remaining groups (61% for sons and 50% for others). Marital status and education level were also an insignificant predictor for explaining burden (all three forms) when controlling for caregiver relationship. We found significant variability in caregivers who recently worked for pay, ranging from 15% in 2011 for spouses (11% in 2015) to 50% of sons in 2011 and 2015, and 32% others in 2011 (and 40% in 2015). All groups reported high levels of care recipient appreciation (88%) and overall general health, with at least 76% of respondents agreeing to feeling well in any given year. Caregiver’s health status was statistically significant even when controlling for caregiver relationship. 

Since health was highly correlated with recent work status and perceived appreciation, we did not include the former into the regression models to avoid multicollinearity. Caregivers reported lower percentages when asked if they had enough time for themselves, though at least 50% of them responded affirmatively, with sons having the lowest overall percentages (57% in 2011 and 52% in 2015), then other caregivers (66% in 2011 and 60% in 2015), followed by husbands or partners (72% in 2011 and 50% 2015). It is noteworthy to highlight the disparaging percentage difference of the latter group between years.

In the caregiving tasks and frequency category, over 50% of sons (54% in 2011 and 51% in 2015) and others (52% in 2011 and 56% in 2015) had *never* helped with personal care versus 40% of spouses in 2011 and 37% of partners in 2015. In fact, only 10% of sons in 2011 (11% in 2015), 14% of other caregivers in 2011 (12% in 2015) and 20% of spouses in 2011 (15% in 2015) had assisted with personal care tasks daily. Caregivers aided with chores at higher rates every day: 46% of spouses, 26% of sons (23% in 2015), and 24% of others, while about a quarter of spouses, sons and others had helped some days in a week, and only 4% of husbands had never helped with chores as compared to 24% of sons in 2011 (23% in 2015) and 27% of others in 2011 (24% in 2015). As for overall caregiving duration, over 92% of spouses, sons and others reported that they had helped for more than a year, 3.5 hours per day given by spouses, four hours per day by sons, and approximately three hours by other caregivers.

In terms of specific tasks, caregivers served as patient advocates by speaking with medical providers, with nearly 46% of spouses in 2011 (37% in 2015), 59% of sons in 2011 (51% in 2015), and 34% of others in 2011 (27% in 2015). Over 70% of spouses, 60% of sons and 40% of others had financially assisted their loved ones with bills or banking. As for physical support, around 20% of spouses, 11% of sons and 15% of other caregivers helped the care recipient get around the house every day of the week in 2011, compared to a few days per week (25% of spouses in 2011 and 22% in 2015, 35% of sons in 2011 and 34% in 2015, and 31% of others in both years), and to rarely or never assisting (44% of spouses in 2011 and 48% in 2015; 43% of sons (in both years), and 41% others in 2011 and 42% in 2015) said they rarely or never helped the elderly with getting around. When asked whether the caregivers had assisted the elderly in leaning weight for support, over 40% of all caregivers said it was inapplicable. Among those caregivers who responded, in 2011 14% spouses, 11% sons and others said they supported every day or most days, whereas in 2015 13% spouses, 12% sons and 12.5% others reported to have supported every day in contrast to 20% spouses, 23% sons and 19% others helped most days. Over 40% caregivers said they supported at least some days, and 35% sons in 2011 and 27% in 2015 and 29% spouses and others in 2011 but 20% spouses in 2015 and 25% others in 2015 said they rarely supported.

Finally, for the caregiver resources category, we noticed that although a high percentage of sons (87% in 2011 and 81% in 2015) and others (79% in 2011 and 76% in 2015) had some family and friends assist with caregiving, only 44% of spouses did in 2011 and 48% in 2015. Furthermore, 10% or fewer spouses, sons and others reported looking for help while caregiving and or receiving any training. 

Using hierarchical logistic regression models, we found that when the caregiver reported having enough time for himself, this was consistently and strongly associated with lower odds of experiencing emotional, financial, and physical burden (Table 3, Table 4 and Table 5) except for other caregivers in 2015 when block 2 and 3 variables were included. Recently working for pay was also linked with lower odds of any burden for all caregivers. In particular, sons experienced a significant reduction in the odds of financial and physical burden in 2011 (Table 4 and Table 5), while spouses showed a somewhat significant decrease in the odds of physical burden in 2015 (Table 5), and others had significantly lower odds for all burdens in both years. Hierarchical model also allowed us to see that working for pay was significant for sons in 2011 only when we didn’t account for blocks 2 and 3. In addition, feeling appreciated by the care recipient was always linked with lower odds of all three burdens (Table 3, Table 4 and Table 5). This association was invariably significant when analyzing the likelihood of emotional strain across all caregivers in 2015 (Table 3), but only for others in 2011. In the financial and physical burden study the feeling of appreciation was significant only for spousal caregivers in 2015 (Table 4 and Table 5) except when variables from blocks 2 and 3 were included in the physical burden (Table 5). 

Assisting with personal care every day increased the odds of experiencing all three burden types (except physical burden experienced by spouses in 2015) as compared to sometimes, rarely or never assisting with personal care (Table 3, Table 4 and Table 5). Unlike personal care frequency, the number of caregiving hours per day was not consistently associated with the odds of experiencing burden. For example, the odds of experiencing burden increased for sons who were providing additional caregiving hours during at least one of the two study years (Table 3, Table 4 and Table 5), while offering additional hours of caregiving was only significantly associated with higher odds of financial burden for spouse caregivers in 2015 (Table 4), and with lower odds of emotional burden for other caregivers in 2011 (Table 3). Having assisted the care recipient for over a year suggested an insignificant association with all three burdens for all caregivers.

All men without family and friends helping with caregiving reported higher odds of these three burden types in 2011 (Table 3, Table 4 and Table 5). In 2015, this relationship was only significant for sons’ emotional burden (Table 3) and the physical burden experienced by other caregivers (Table 5). Seeking for caregiving assistance was also related to higher odds of experiencing burden across all caregivers. This pattern was significant for the odds of emotional burden of spouses and sons in 2011, and for other caregivers in 2015 (Table 3). Similarly, sons in 2011 and 2015 and other caregivers in 2011 had a significant increase in the odds of financial burden when looking for caregiving assistance (Table 4). Spouses who received training reported a significant rise in the odds of physical burden in 2011 (Table 5), while sons who had training also reported a significant increase in the odds of emotional burden in the same year (Table 3). Other caregivers receiving training actually reported lower chances of experiencing all forms of burden, but the only significant relationship for this group was associated with higher odds of physical stress (Table 5). 

In sum, working for pay last week, having enough time for himself, and feeling appreciated by the care recipient were associated with lower odds of experiencing all burden types across the three groups, while engaging more frequently in personal care was related to higher chances of experiencing any form of burden for all caregivers. In addition, spouses and sons offering more hours of caregiving felt more strained, but this predictor actually reduced the likelihood of experiencing burden for other caregivers. Furthermore, we noticed changes in the contribution of block 1 variables when either block 2 or both block 2 and 3 variables were added, while contribution of block 2 variables remained consistent after addition of variables in block 3. Having friends and family assist with caregiving was linked to a lower chance of experiencing burden, though only significant for the emotional stress of son caregivers and the physical stress of other caregivers. Seeking for caregiving support and receiving training were generally linked with higher chances of experiencing burden—especially whenever the association was significant—perhaps suggesting that by the time caregivers requested help, they were already burdened.

## 4. Discussion

Unmet service needs increase caregiver burden [44] and several resources can alleviate this strain while increasing competence, including personal networks, respite time, information access, and skill development. Our study revealed that, controlling for relationship, all caregivers experienced emotional, financial and physical burden across both years and higher odds of emotional burden compared to financial and physical strain. Sons reported a higher chance of emotional and financial stress among all caregivers, while husbands showed a higher likelihood of experiencing physical burden. These results highlight the importance of acknowledging caregiving support policies that include relationship with the care recipient and that focus on specific subgroups, such as sons who may be trying to balance personal, family, and work issues while simultaneously taking care of their parent(s). 

Because there is no one-size-fits-all resource package for all caregivers, it is important to tailor these services according to the caregiver’s needs and personal characteristics [70]. For example, husbands may need more emotional support and to communicate with other spousal caregivers in support groups, while sons may find short-term workshops more fitting to their preferential needs of skill acquisition over formal support systems [47,71,72]. Such dissimilarities reflect relationship differences between the care recipient and the caregiver given that husbands are looking after their life partner and experiencing life changes themselves, while sons and other groups may be bonded by filial obligations but feel less overall emotional intensity [19,72]. Men caregivers are not a monolithic group providing care in a similar way or experiencing similar types of caregiver burden, nor do they use support systems with the same intensity. As such, each current and potential caregiver subgroup requires diverse policies supporting their roles and tasks. 

Despite lower participation rates in personal care compared to other tasks or to women caregivers, men have increased their role in assisting with personal care activities [73]. Sometimes caregivers do not engage in a particular activity (cooking, cleaning, or bathing an aging parent) because they are unable to do it, not necessarily because they have gendered preconceptions about it. Regardless of participation rates, assisting with personal care was one of the most significant contributors of increasing the odds of all three burden types for most caregivers. While we were aware of the impact of personal care tasks on all caregivers in general and male caregivers in particular, we were particularly struck by how the likelihood of experiencing any type of burden diminished when these caregivers were still conducting these duties, but not on a daily basis. Caregivers need to be more proactive in seeking adequate training which empowers them to perform increasingly complicated and delicate tasks, and to protect themselves from the additional stress that occurs when one feels unprepared and overwhelmed. Moreover, policymakers need to aggressively target male caregivers who are increasingly performing these tasks and to funnel or reallocate resources providing broader respite relief to caregivers who assist with personal care so that they are not the only ones dealing with these tasks.

Respite care—the temporary care usually provided to the frail elder by a public or private group to allow relief for the caregiver—can be an effective supportive measure and is a key burden suppressant, though is unfortunately not always accessible [74]. Caregivers should try to give time to themselves guilt-free for their own well-being and in order to continue providing care to their loved ones, as becoming a caregiver does not mean that the person should neglect their own health. But the nature of the role and the existing weak support systems contribute to the caregiver losing himself and experiencing significant burden. Our results consistently suggested that those caregivers with more time for themselves unequivocally had lower odds of experiencing burden, and that having family and friends assist the caregiver diminished the chances of these strains because they prevent the caregiver from feeling overwhelmed and isolated [75,76]. Even perceived family support can significantly reduce psychological distress [77]. Not all caregivers have supportive personal networks and men have even smaller networks than women do [56], so it is very important to prevent caregiver isolation and to help supplement the caregiving that they do.

We were also pleased to notice and capture that close to 88% of the caregiver sample felt appreciated by their care recipients, suggesting strong relationship bonds between them even during a difficult time and process. To reaffirm the importance of strong and healthy family bonds, our results confirmed that all caregiver types who did not feel appreciated by their care recipients significantly reported higher odds of emotional, financial, and physical burden.

Only a small percentage of male caregivers looked for any help or skill development, including support groups, respite care, training, or information. In fact, men and women caregivers under-utilize available services even when they claim that they will use them [14,78], and unused services occurred more frequently among caregivers providing intensive care. So, by the time they decide to seek help, caregivers may be extremely burdened and overwhelmed. It would be ideal if caregivers utilized these services when starting this role and updated them, even for the most experienced caregivers, especially because this role will evolve with the needs of the care recipient [79]. Steps should be taken to increase access for caregivers who provide intensive care and who may also be constrained by geographic variation in the supply of service providers [80,81]. Policy makers should reallocate resources towards support that caregivers find most useful and with (potentially) higher utilization rates [81]. For example, in Spain the informal caregiver is recognized as the center of care provision at home, but is also fully integrated into the overall system of social protection, becoming the beneficiary of financial compensation and support programs, such as training courses, respite opportunities, and caregiver leave, to ultimately help equalize men and women’s paid work and family duties [82,83]. 

## 5. Conclusions

Though family and friends are the largest group of caregivers in the United States, they are strained and our nation does little to support them [1]. In an aging world requiring everyone to be a caregiver, it is urgent to attract and to maintain a larger, more sustainable and less burdened pool. Social and health care trends are placing increasing pressures on men in their families and communities to take on more caregiving roles in the future. As cultural and gender paradigms shift towards a broader concept of what caregiving is and who engages in this valuable activity, we need to take advantage of this shift and to provide more encompassing, hands-on training and support to people who can become competent caregivers. 

Our research assessed emotional, financial, and physical burden that male caregivers experienced based on their relationship with the elderly. We found that all caregivers had different types and levels of strain. Decomposing caregiving burden into emotional, financial, and physical allowed us to detect trends among different male caregivers based on their relationship with the recipient. Support (such as help with skill development, respite care, training, or information) which could significantly improve the quality of caregiving, has been consistently underutilized irrespective of relationship and caregiving levels. Both primary and secondary caregivers experience burden, and it is important to help alleviate it. No caregiver, man or woman, can provide such an intensive task alone, so we need to pay attention to caregiver well-being because they significantly contribute to societal welfare. Thus, we need a community of care for aging individuals and for their caregivers to strengthen our social safety net. 

## Figures and Tables

**Table 1:** Predictor variables for emotional, financial, and physical burden by blocks and sample sizes ($n_{2011},n_{2015}$) for each caregiver group in 2011 and 2015.

Blocks	Predictor Variables (Variable Type)	Sample Sizes		
		Spouse $\left(n_{2011},\;n_{2015}\right)$	Son $\left(n_{2011},n_{2015}\right)$	Other $\left(n_{2011},n_{2015}\right)$
**I**	Have you worked for pay last week? (binary: yes or no)	(169, 198)	(306, 330)	(190, 182)
	Do you have time for yourself? (binary: yes or no)	(169, 197)	(309, 331)	(191, 182)
	Does elderly appreciate you? (binary: yes or no)	(166, 191)	(306, 329)	(185, 178)
**II**	How often they helped with personal care (categorical with five levels: every day, most days, some days, rarely and never)	(172, 198)	(310, 336)	(190, 183)
	Number of hours helped per day (hours)	(172, 198)	(310, 337)	(191, 183)
	Have you helped longer than a year (binary: yes or no)	(169, 198)	(310, 337)	(190, 183)
**III**	Do you have family and friends to help with caregiver? (binary: yes or no)	(171, 197)	(309, 334)	(191, 183)
	Have you ever looked for help? (binary: yes or no)	(170, 198)	(307, 331)	(190, 182)
	Have you ever received training/help (binary: yes or no)	(169, 198)	(309, 334)	(191, 183)

**Table 2:** Log odds ratio coefficients (*p*-values and sample size) from regressing caregivers’ emotional, financial, and physical difficulty on their relationship to the elderly in 2011 and 2015 with statistically significant coefficients (1%, 5% and 10% significance levels) highlighted.

Response (Variable Type)	2011		2015	
	Spouse	Son	Spouse	Son
**Emotional Burden** (binary: yes or no)	0.34 (0.146, 171)	0.88 (0.00001, 307)	0.29 (0.207, 198)	0.72 (0.00047, 335)
**Financial Burden** (binary: yes or no)	−0.01 (0.964, 170)	0.47 (0.039, 305)	−0.02 (0.950, 197)	0.11 (0.672, 334)
**Physical Burden** (binary: yes or no)	0.54 (0.056, 169)	0.36 (0.159, 309)	0.57 (0.052, 198)	0.10 (0.724, 335)

**Table 3:** Data from the 2011 and 2015 hierarchical logistic regression log odds ratio coefficients (*p*-values) for caregivers’ emotional burden for predictor blocks 1–3 with statistically significant coefficients highlighted.

Predictor/Response	Spouse	Son	Other	Spouse	Son	Other
	2011			2015		
**Worked for pay last week (Y/N)**						
Block 1	−0.43 (0.37)	0.41 (0.09)	−0.25 (0.50)	0.20 (0.73)	−0.16 (0.51)	0.61 (0.11)
Blocks 1 and 2	−0.80 (0.17)	0.25 (0.35)	0.05 (0.90)	0.06 (0.92)	−0.41 (0.12)	0.64 (0.13)
Blocks 1, 2, and 3	−0.47 (0.47)	0.26 (0.35)	0.04 (0.93)	0.15 (0.82)	−0.42 (0.12)	0.66 (0.13)
**Time for yourself (Y/N)**						
Block 1	0.87 (0.02)	1.23 (0.00)	0.86 (0.02)	1.17 (0.00)	0.89 (0.00)	0.74 (0.05)
Blocks 1 and 2	0.79 (0.06)	1.15 (0.00)	1.03 (0.01)	1.06 (0.01)	0.75 (0.00)	0.63 (0.12)
Blocks 1, 2, and 3	0.73 (0.09)	1.08 (0.00)	1.05 (0.01)	1.07 (0.01)	0.74 (0.00)	0.55 (0.19)
**Elderly appreciates you (Y/N)**						
Block 1	0.54 (0.27)	0.61 (0.19)	1.90 (0.00)	1.31 (0.03)	1.54 (0.00)	1.09 (0.04)
Blocks 1 and 2	0.19 (0.75)	0.41 (0.41)	2.62 (0.00)	1.57 (0.02)	1.75 (0.00)	0.93 (0.14)
Blocks 1, 2, and 3	0.15 (0.80)	0.24 (0.65)	2.71 (0.00)	1.46 (0.04)	1.72 (0.00)	0.85 (0.20)
**Personal care Frequency (Most days)**						
Blocks 1 and 2	0.49 (0.54)	−0.48 (0.39)	0.26 (0.77)	0.50 (0.45)	0.63 (0.31)	0.18 (0.86)
Blocks 1, 2, and 3	0.28 (0.74)	−0.45 (0.55)	0.25 (0.78)	−0.54 (0.42)	0.57 (0.37)	−0.13 (0.91)
**Personal care Frequency (Some days)**						
Blocks 1 and 2	−0.08 (0.90)	0.89 (0.10)	−0.36 (0.61)	−0.53 (0.35)	0.14 (0.77)	−0.66 (0.35)
Blocks 1, 2, and 3	−0.28 (0.64)	−0.73 (0.22)	−0.27 (0.70)	−0.60 (0.30)	0.08 (0.87)	−0.59 (0.43)
**Personal care Frequency (Rarely)**						
Blocks 1 and 2	−0.34 (0.64)	−0.43 (0.45)	0.26 (0.69)	−0.89 (0.15)	−0.78 (0.14)	−1.73 (0.04)
Blocks 1, 2, and 3	−0.50 (0.50)	−0.14 (0.83)	0.29 (0.66)	−0.83 (0.18)	−1.07 (0.05)	−1.57 (0.08)
**Personal care Frequency (Never)**						
Blocks 1 and 2	−1.15 (0.04)	−0.95 (0.05)	−1.26 (0.03)	−0.89 (0.09)	−0.19 (0.68)	−1.42 (0.03)
Blocks 1, 2, and 3	−1.21 (0.03)	−0.77 (0.16)	−1.24 (0.03)	−0.85 (0.12)	−0.32 (0.50)	−1.34 (0.06)
**Hours helped per day (hrs)**						
Blocks 1 and 2	−0.02 (0.71)	0.09 (0.01)	−0.18 (0.06)	0.03 (0.55)	0.03 (0.40)	−0.06 (0.42)
Blocks 1, 2, and 3	−0.05 (0.41)	0.08 (0.03)	−0.19 (0.05)	0.02 (0.60)	0.03 (0.43)	−0.09 (0.33)
**Helped over a year (Y/N)**						
Blocks 1 and 2	0.29 (0.71)	0.84 (0.23)	−0.56 (0.55)	0.23 (0.72)	0.46 (0.43)	−15.32 (0.99) ^1^
Blocks 1, 2, and 3	0.25 (0.75)	0.48 (0.51)	−0.55 (0.56)	0.27 (0.68)	0.50 (0.42)	−15.14 (0.99) ^1^
**Family and friends help caregiver (Y/N)**						
Blocks 1, 2, and 3	0.43 (0.29)	1.50 (0.00)	0.11 (0.84)	−0.33 (0.38)	0.99 (0.00)	−0.29 (0.59)
**Looked for help (Y/N)**						
Blocks 1, 2, and 3	−2.50 (0.03)	−0.79 (0.09)	−0.16 (0.86)	−0.84 (0.36)	−0.46 (0.27)	−1.38 (0.06)
**Received training (Y/N)**						
Blocks 1, 2, and 3	−0.91 (0.38)	−1.00 (0.07)	0.79 (0.54)	0.00 (1.00)	0.26 (0.60)	0.10 (0.92)

^1^ In the emotional burden category, the number of other caregivers who said they have helped over a year in 2015 was only 3.8%; hence the computed log odds are invalid.

**Table 4:** Data from the 2011 and 2015 hierarchical logistic regression log odds ratio coefficients (*p*-values) for caregivers’ financial burden for predictor blocks 1–3 with statistically significant coefficients highlighted.

Predictor/Response	Spouse	Son	Other		Spouse	Son	Other
	2011				2015		
**Worked for pay last week (Y/N)**							
Block 1	0.34 (0.62)		0.68 (0.01)	−0.21 (0.59)	−0.02 (0.98)	0.39 (0.17)	0.74 (0.10)
Blocks 1 and 2	0.85 (0.34)		0.57 (0.05)	−0.22 (0.60)	−0.12 (0.87)	0.20 (0.53)	0.71 (0.13)
Blocks 1, 2, and 3	0.87 (0.37)		0.63 (0.04)	−0.19 (0.66)	−0.16 (0.82)	0.26 (0.42)	0.77 (0.10)
**Time for yourself (Y/N)**							
Block 1	1.56 (0.00)		1.19 (0.00)	1.05 (0.01)	1.15 (0.01)	0.70 (0.02)	0.92 (0.03)
Blocks 1 and 2	1.51 (0.00)		1.11 (0.00)	1.12 (0.01)	0.94 (0.06)	0.60 (0.06)	0.87 (0.05)
Blocks 1, 2, and 3	1.57 (0.00)		1.05 (0.00)	1.12 (0.01)	0.90 (0.08)	0.62 (0.06)	0.87 (0.06)
**Elderly appreciates you (Y/N)**							
Block 1	0.04 (0.95)		−0.37 (0.50)	0.45 (0.50)	0.90 (0.16)	0.16 (0.71)	0.35 (0.57)
Blocks 1 and 2	−0.67 (0.46)		−0.37 (0.53)	1.07 (0.14)	1.24 (0.08)	0.40 (0.39)	0.96 (0.16)
Blocks 1, 2, and 3	−0.82 (0.38)		−0.35 (0.54)	1.01 (0.19)	1.22 (0.11)	0.40 (0.40)	0.89 (0.20)
**Personal care Frequency (Most days)**							
Blocks 1 and 2	0.48 (0.59)		−0.75 (0.26)	−0.59 (0.50)	−0.25 (0.75)	−0.83 (0.24)	−0.52 (0.68)
Blocks 1, 2, and 3	0.41 (0.65)		−0.88 (0.23)	−0.76 (0.41)	−0.31 (0.70)	−0.83 (0.26)	−0.51 (0.70)
**Personal care Frequency (Some days)**							
Blocks 1 and 2	−0.24 (0.72)		−1.24 (0.02)	−0.29 (0.68)	−0.60 (0.38)	−0.52 (0.33)	−0.09 (0.91)
Blocks 1, 2, and 3	−0.24 (0.72)		−1.08 (0.06)	−0.40 (0.59)	−0.66 (0.35)	−0.46 (0.41)	−0.12 (0.88)
**Personal care Frequency (Rarely)**							
Blocks 1 and 2	−1.58 (0.19)		−0.60 (0.28)	0.17 (0.80)	−0.89 (0.27)	−0.34 (0.54)	−0.53 (0.51)
Blocks 1, 2, and 3	−1.61 (0.18)		−0.29 (0.62)	0.25 (0.72)	−0.95 (0.24)	−0.37 (0.53)	−0.67 (0.42)
**Personal care Frequency (Never)**							
Blocks 1 and 2	−1.59 (0.03)		−1.06 (0.02)	−0.86 (0.15)	−0.24 (0.69)	−0.88 (0.08)	−0.99 (0.16)
Blocks 1, 2, and 3	−1.63 (0.03)		−0.80 (0.11)	−1.00 (0.10)	−0.30 (0.63)	−0.76 (0.15)	−1.01 (0.17)
**Hours helped per day**							
Blocks 1 and 2	−0.01 (0.81)		0.06 (0.08)	0.05 (0.38)	0.04 (0.36)	0.07 (0.05)	0.03 (0.61)
Blocks 1, 2, and 3	−0.02 (0.75)		0.05 (0.10)	0.01 (0.91)	0.04 (0.38)	0.08 (0.03)	0.04 (0.52)
**Helped over a year (Y/N)**							
Blocks 1 and 2	−15.48 (0.99) ^2^		−0.28 (0.75)	−0.98 (0.38)	−16.59 (0.99) ^2^	0.32 (0.62)	0.29 (0.80)
Blocks 1, 2, and 3	−15.53 (0.99) ^2^		−0.47 (0.62)	−0.90 (0.43)	−16.64 (0.99) ^2^	0.44 (0.50)	0.52 (0.66)
**Family and friends help caregiver (Y/N) **							
Blocks 1, 2, and 3	0.37 (0.50)		0.24 (0.56)	0.45 (0.40)	0.05 (0.91)	0.02 (0.97)	−0.63 (0.32)
**Looked for help (Y/N)**							
Blocks 1, 2, and 3	−0.01 (1.00)		−0.99 (0.02)	−1.71 (0.03)	−0.19 (0.85)	−1.17 (0.00)	0.49 (0.59)
**Received training (Y/N)**							
Blocks 1, 2, and 3	0.18 (0.90)		−0.46 (0.37)	0.04 (0.97)	−0.60 (0.51)	0.12 (0.84)	0.26 (0.79)

^2^ In the financial burden category, the number of spouse caregivers who said they have helped over a year in 2011 and 2015 were only 6% and 9% respectively; hence the computed log odds are invalid.

**Table 5:** Data from the 2011 and 2015 hierarchical logistic regression log odds ratio coefficients (*p*-values) for caregivers’ physical burden for predictor blocks 1–3 with statistically significant coefficients highlighted.

Predictor/Response	Spouse	Son	Other	Spouse	Son	Other
	2011			2015		
**Worked for pay last week (Y/N)**						
Block 1	0.61 (0.37)	0.69 (0.03)	0.28 (0.55)	1.75 (0.10)	0.67 (0.05)	1.68 (0.01)
Blocks 1 and 2	0.38 (0.59)	0.55 (0.11)	0.35 (0.48)	1.52 (0.17)	0.25 (0.52)	1.39 (0.05)
Blocks 1, 2, and 3	1.14 (0.20)	0.64 (0.07)	0.33 (0.53)	1.46 (0.19)	0.28 (0.46)	1.38 (0.06)
**Time for yourself (Y/N)**						
Block 1	1.14 (0.00)	0.88 (0.00)	0.99 (0.03)	1.63 (0.00)	1.01 (0.01)	1.38 (0.01)
Blocks 1 and 2	0.91 (0.04)	0.67 (0.00)	0.92 (0.05)	1.53 (0.00)	0.68 (0.07)	0.93 (0.13)
Blocks 1, 2, and 3	0.84 (0.08)	0.63 (0.07)	0.82 (0.09)	1.58 (0.00)	0.66 (0.09)	0.89 (0.15)
**Elderly appreciates you (Y/N)**						
Block 1	0.09 (0.88)	0.75 (0.13)	0.41 (0.56)	1.10 (0.09)	0.00 (0.99)	0.97 (0.15)
Blocks 1 and 2	−0.17 (0.81)	0.87 (0.12)	0.44 (0.61)	1.31 (0.08)	0.32 (0.57)	1.11 (0.26)
Blocks 1, 2, and 3	0.00 (1.00)	0.89 (0.11)	−0.01 (0.99)	0.92 (0.27)	0.39 (0.49)	1.05 (0.29)
**Personal care Frequency (Most days)**						
Blocks 1 and 2	0.41 (0.63)	0.06 (0.92)	−0.24 (0.81)	−0.50 (0.54)	−0.56 (0.41)	−16.16 (0.99)
Blocks 1, 2, and 3	0.63 (0.51)	−0.01 (0.99)	−0.05 (0.96)	−0.63 (0.46)	−0.51 (0.45)	−16.12 (0.99)
**Personal care Frequency (Some days)**						
Blocks 1 and 2	−0.08 (0.90)	−1.60 (0.01)	−0.19 (0.80)	−0.43 (0.53)	−0.95 (0.10)	−0.06 (0.94)
Blocks 1, 2, and 3	−0.22 (0.75)	−1.49 (0.02)	−0.31 (0.69)	−0.37 (0.60)	−0.90 (0.12)	−0.03 (0.97)
**Personal care Frequency (Rarely)**						
Blocks 1 and 2	−0.51 (0.59)	−0.84 (0.14)	−0.70 (0.39)	−1.20 (0.19)	−1.30 (0.04)	−1.92 (0.12)
Blocks 1, 2, and 3	−0.52 (0.60)	−0.66 (0.28)	−0.84 (0.31)	−1.35 (0.16)	−1.18 (0.07)	−1.88 (0.14)
**Personal care Frequency (Never)**						
Blocks 1 and 2	−0.45 (0.47)	−1.70 (0.00)	−0.80 (0.21)	0.22 (0.71)	−1.72 (0.00)	−2.00 (0.02)
Blocks 1, 2, and 3	−0.26 (0.69)	−1.54 (0.00)	−1.14 (0.09)	0.10 (0.88)	−1.61 (0.00)	−1.96 (0.03)
**Hours helped per day**						
Blocks 1 and 2	0.06 (0.27)	0.02 (0.60)	−0.04 (0.60)	0.07 (0.10)	0.06 (0.09)	−0.01 (0.90)
Blocks 1, 2, and 3	0.05 (0.34)	0.02 (0.63)	−0.04 (0.63)	0.08 (0.09)	0.07 (0.07)	−0.01 (0.90)
**Helped over a year (Y/N)**						
Blocks 1 and 2	−15.94 (0.99) 3	0.32 (0.72)	−15.79 (0.99) 3	−0.89 (0.42)	0.44 (0.54)	1.01 (0.42)
Blocks 1, 2, and 3	−15.85 (0.99) 3	0.30 (0.74)	−16.41 (0.99) 3	−0.84 (0.45)	0.48 (0.51)	1.10 (0.39)
**Family and friends help caregiver (Y/N)**						
Blocks 1, 2, and 3	−0.43 (0.36)	0.10 (0.83)	1.00 (0.08)	0.67 (0.17)	−0.35 (0.51)	−0.20 (0.80)
**Looked for help (Y/N)**						
Blocks 1, 2, and 3	−1.44 (0.14)	−0.43 (0.37)	0.14 (0.89)	−1.03 (0.28)	−0.35 (0.52)	−0.26 (0.79)
**Received training (Y/N)**						
Blocks 1, 2, and 3	−2.30 (0.05)	−0.19 (0.74)	−1.79 (0.11)	−0.13 (0.89)	−0.30 (0.62)	0.14 (0.91)

^3^ In the physical burden category, the number of spouse and other caregivers who said they have helped over a year in 2011 were only 5.9% and 8%, respectively; hence the computed log odds are invalid.
